# Improving outpatient care for heart failure through digital innovation: a feasibility study

**DOI:** 10.1186/s40814-022-01206-w

**Published:** 2022-11-30

**Authors:** David O. Arnar, Saemundur J. Oddsson, Thrudur Gunnarsdottir, Gudbjorg Jona Gudlaugsdottir, Elias Freyr Gudmundsson, Audur Ketilsdóttir, Hulda Halldorsdottir, Thordis Jona Hrafnkelsdottir, Hallur Hallsson, Maria L. Amundadottir, Tryggvi Thorgeirsson

**Affiliations:** 1 Cardiovascular Centre, Landspitali – The National University Hospital of Iceland, Hringbraut, 101 Reykjavik, Iceland; 2 Faculty of Medicine, University of Iceland, Reykjavik, Iceland; 3 Sidekick Health Digital Therapeutics, Kopavogur, Iceland

**Keywords:** Clinical outcome, Digital solution, Feasibility study, Heart failure, Lifestyle-change, Remote monitoring, Self-care

## Abstract

**Background:**

Heart failure (HF) affects over 26 million people worldwide. Multidisciplinary management strategies that include symptom monitoring and patient self-care support reduce HF hospitalization and mortality rates. Ideally, HF follow-up and self-care support includes lifestyle-change recommendations and remote monitoring of weight and HF symptoms. Providing these via a digital solution may be ideal for improving HF disease outcomes and reducing the burden on providers and healthcare systems. This study’s main objective was to assess the feasibility of a digital solution including remote monitoring, lifestyle-change, and self-care support for HF outpatients in Iceland.

**Methods:**

Twenty HF patients (mean age 57.5 years, 80% males) participated in an 8-week study. They were provided with a digital solution (SK-141), including lifestyle-change and disease self-care support, a remote symptom monitoring system, and a secure messaging platform between healthcare providers and patients. This feasibility study aimed to assess patient acceptability of this new intervention, retention rate, and to evaluate trends in clinical outcomes. To assess the acceptability of SK-141, participants completed a questionnaire about their experience after the 8-week study. Participants performed daily assigned activities (missions), including self-reporting symptoms. Clinical outcomes were assessed with the Hospital Anxiety and Depression Scale and the Kansas City Cardiomyopathy Questionnaire at the study's beginning and end with an online survey.

**Results:**

Of the 24 patients invited, 20 were elected to participate. The retention rate of participants throughout the 8-week period was high (80%). At the end of the 8 weeks, thirteen participants completed a questionnaire about their experience and acceptability of the SK-141. They rated their experience positively including on questions whether they would recommend the solution to others (6.8 on a scale of 1–7), whether the solution had improved their life and well-being (5.7 on a scale of 1–7), and whether it was user friendly (5.5 on a scale of 1–7). Many of the clinical parameters studied exhibited a promising trend towards improvement over the 8-week period.

**Conclusion:**

The digital solution, SK-141, was very acceptable to patients and also showed promising clinical results in this small feasibility study. These results encourage us to conduct a longer, more extensive, adequately powered, randomized-controlled study to assess whether this digital solution can improve the quality of life and clinical outcomes among HF patients.

**Supplementary Information:**

The online version contains supplementary material available at 10.1186/s40814-022-01206-w.

## Key messages regarding feasibility

- Before this study, we were uncertain of the acceptability of a digital solution in this patient population and whether the solution might show promise for positively impacting patients' health and well-being.
- The participants rated the solution highly and considered it user-friendly. Additionally, we observed some improvements in health-related and clinical variables, although few were statistically significant in this small study.
- Based on the acceptability and promising observations from clinical variables from this feasibility study, a large randomized-controlled study is planned.

## Introduction

Heart failure (HF) is an abnormality of the cardiac structure and function leading to failure of the heart to deliver oxygen to tissues at the required rate [1]. HF is a serious condition with an estimated prevalence rate in developed countries of 4.2% in the general adult population and 11.8% among people ≥60 years of age [2]. HF affects about 26 million people worldwide, creating a significant burden on healthcare providers and patients [3]. HF also presents a heavy economic burden; for instance, the total cost of HF in the USA alone was estimated to be $30.7 billion in 2012 and is expected to increase to $69.7 billion by 2030 [4]. HF hospital admission and readmission rates, and mortality rates, remain high despite advances in evidence-based medical and device therapy [5].

Large prospective cohort studies, such as the one including over 64,000 people with 13 years of follow-up, have shown that healthy lifestyle factors, such as non-smoking, physical activity, a healthy body mass index, and a healthy diet, can greatly decrease the risk of developing HF [6]. In patients with pre-existing HF, hospital (re)admission, morbidity and mortality can also be modified by lifestyle changes. For example, improved self-care decreased the length of HF-related hospitalizations [7], while a combination of lifestyle changes reduced both the number and length of hospital admissions among HF patients [8]. Also, higher exercise training workloads lowered the risk of all-cause mortality and HF hospitalization [9], while increased physical activity reduced the risk of atrial fibrillation in HF patients [10].

Given the significant morbidity in patients, the high cost and burden on health care, and the many modifiable HF risk factors that are potential targets for intervention, there has been increasing interest in developing multidisciplinary strategies to improve outcomes for HF patients. A systematic review of 29 clinical trials found that multidisciplinary management strategies that included specialized follow-up reduced mortality, all-cause hospitalization, and HF hospitalization. Multidisciplinary management strategies that included support for improving patient self-care activities reduced all-cause hospitalization and HF hospitalization. In addition, multidisciplinary management strategies proved to be cost-saving [11].

However, multidisciplinary management strategies with specialized follow-up and a focus on improving patient self-care are complex and may require increased patient support and healthcare resources. Non-invasive digital technologies (remote medical care or telemedicine) are uniquely positioned to provide such support in a cost-effective, real-time manner [12, 13]. Because of the widespread ownership of smartphones, digital interventions hold unique promise for delivering specialized follow-up and support to large numbers of people in a format that is readily accessible at almost any time. Approximately two-thirds of the world‘s population owns a smartphone [14], and in 2019 there were 204 billion app downloads [15].

Digital interventions for HF patients are relatively new, and their effectiveness has not yet been studied in large or longitudinal trials. However, small studies have been performed to determine whether various types of remote medical care are effective in improving self-care behavior in HF patients. A recent systematic review of twelve of these studies shows that this is indeed the case for some of the studies, although no clinically significant improvements were found compared to control groups in five studies [16]. The delivery of remote medical care in these studies was highly variable, and better design of these solutions may improve patients' experience and hence improve HF outcomes [17]. One design component with the potential to improve outcomes is the remote monitoring of HF symptoms. Through remote symptom monitoring, early signs of symptom worsening could trigger alerts to be acted upon by patients and providers to avoid acute decompensation and hospitalization.

The current study aimed to assess the feasibility and preliminary effectiveness of a new digital solution (SK-141), that was specifically developed for use by HF patients, with the possibility of conducting a larger randomized study. This solution included a remote symptom monitoring system, lifestyle-change and disease self-care support, and a secure messaging platform between healthcare providers and patients to facilitate improved care and ultimately reduce hospital readmission rates and HF disease burden.

## Methods

### Objectives

This feasibility study aimed to assess patient acceptability of this new intervention, recruitment potential among patients in the HF clinic at Landspitali – The National University Hospital of Iceland, retention rate and engagement with the digital solution over 8 weeks, and evaluate trends in clinical outcomes.

### Participants

Twenty-four consecutive patients who visited the outpatient HF clinic, and fulfilled the eligibility criteria, were invited to participate in an 8-week study consisting of the newly developed digital solution delivered via the Sidekick Health digital platform (Sidekick Health, https://sidekickhealth.com/) during the summer of 2020. HF patients that provided written informed consent were enrolled in the study. Eligibility criteria were age 18–80 years, a diagnosis of heart failure with a reduced ejection fraction, and owning a smartphone or a tablet. We chose patients with a reduced ejection fraction as the group was intended to be rather small and we wanted to have it as uniform as possible. The Icelandic Data Protection Authority approved the study (license number: VSN-21-001), which conforms with the declaration of Helsinki principles.

### The SK-141 solution

**Fig. 1:**
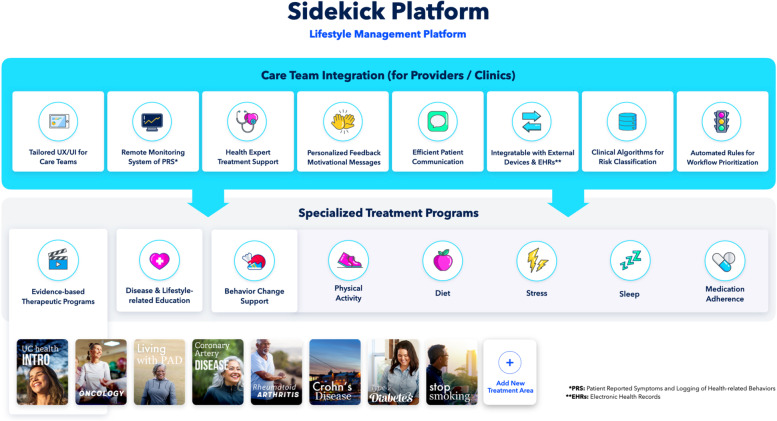
The Sidekick Platform for lifestyle management

A digital solution including a remote symptom monitoring system, lifestyle-change and disease self-care support, and a secure messaging platform between healthcare providers and patients, was developed by a digital healthcare company, Sidekick Health, in collaboration with HF experts at the HF Clinic of the National University Hospital in Reykjavik, Iceland. Sidekick Health has a comprehensive and gamified digital platform for delivering digital therapeutic solutions [18, 19], including the HF solution (SK-141) (Fig. 1). The Sidekick digital platform is designed to modify lifestyle-related behaviors in four main categories: nutrition (food), physical activity (movement), stress management and psychoeducation (mind), and health (tracking of medications, supplements, and health-related outcomes). Participants are assigned gamified tasks (daily missions), and by completing those tasks they accumulate points that provide virtual altruistic rewards. On average the educational videos were approximately 2–3 min in length and participants are expected to spend about 15 min a day in the app if they complete all missions.

**Fig. 2:**
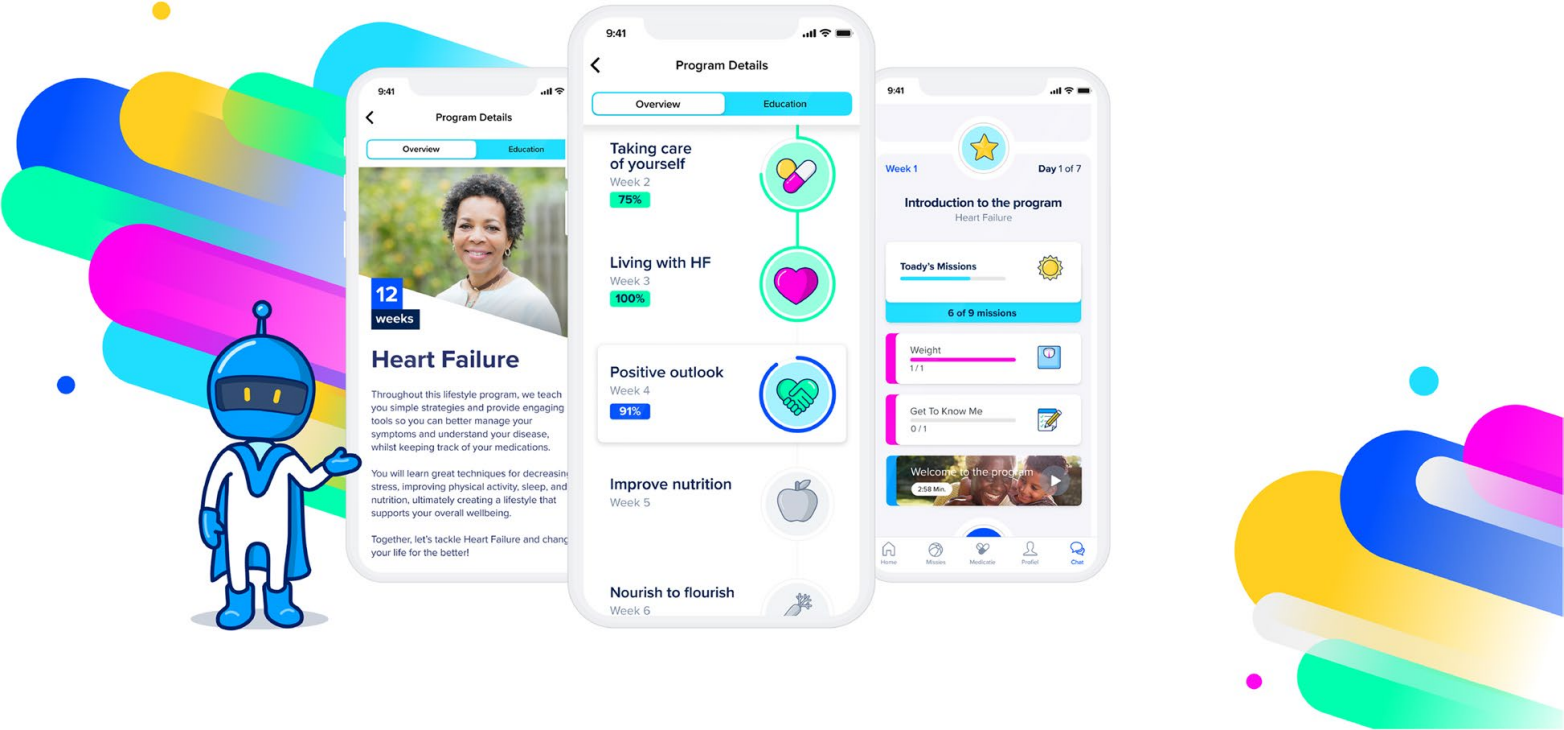
The SK-141 digital solution patient interface

The lifestyle-change and disease-care support in SK-141 included daily missions assigned within the Sidekick platform such as setting goals and logging lifestyle- or disease-care-related behaviors, watching short educational videos on lifestyle- and disease-related topics such as exercise, diet, sleep, meditation, behavioral modification methods, and doing mindfulness-based exercises. Also included were weekly feedback messages from nurses on changes in behaviors logged within the digital platform and recommendations for the upcoming week (Fig. 2). The app is currently available in Icelandic and English.

**Fig. 3:**
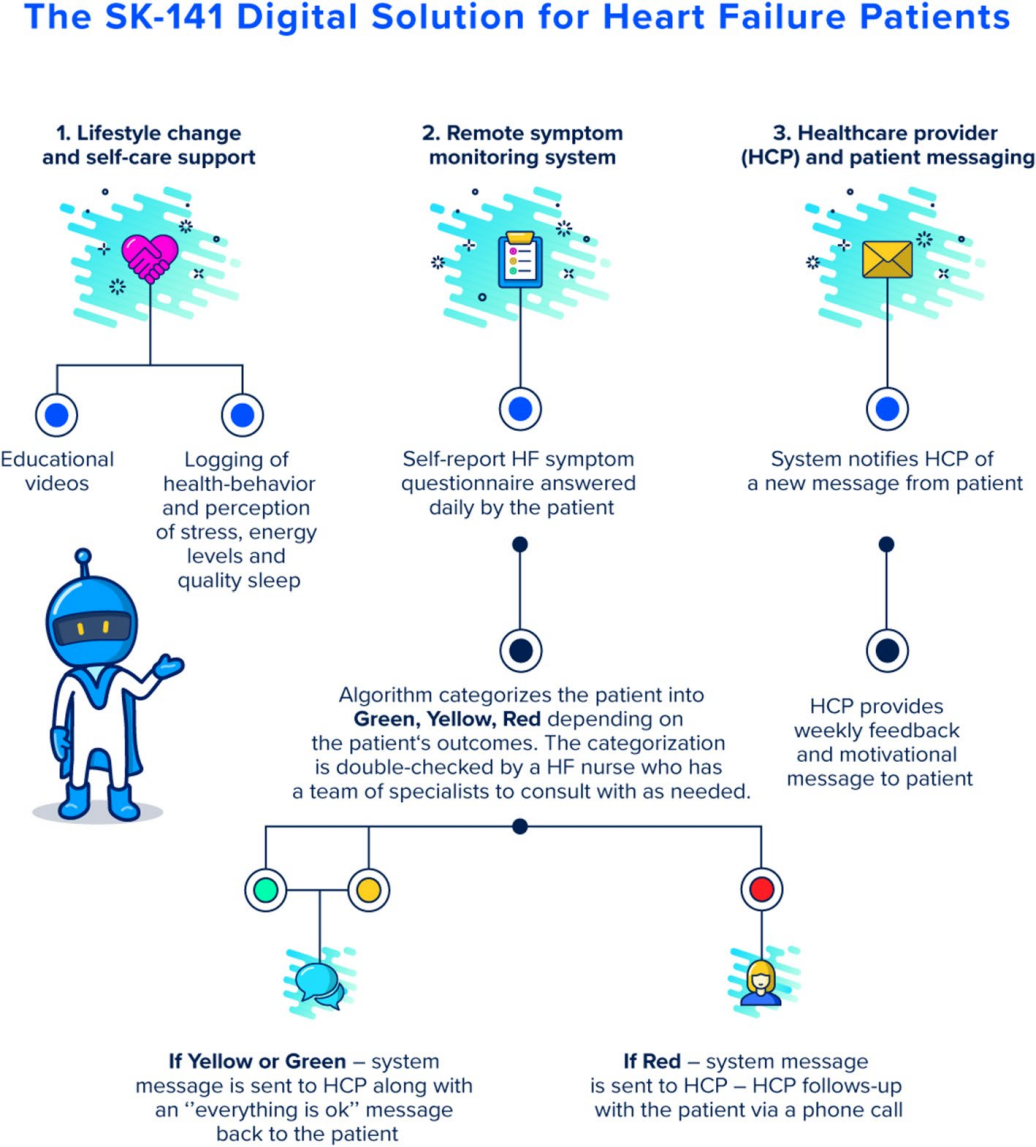
The SK-141 digital solution for heart failure patients

The remote symptom monitoring system included daily questions about changes in weight and HF symptoms. Participants’ responses were weighted, and an algorithm suggested a three-tiered, color-coded (green, yellow, red) risk classification that determined the required response (Fig. 3). A nurse evaluated and confirmed the algorithm's classification and provided feedback and support to participants weekly or more frequently if indicated by the system. Messages were delivered via a chat function within the digital platform or, if a patient was classified as high risk (red), via a direct phone call to the patient.

### Outcomes

Acceptability was assessed based on a number of variables. In addition to a questionnaire completed by patients after the completion of the study, the percentage of individuals enrolling in the study of those invited, and the retention rate of those enrolled were also taken into account. Although the study was small, we also attempted to assess trends in a variety of clinical outcomes with regards to help us determine sample size calculations should a larger randomized clinical trial be deemed feasible.

Participants reported their daily energy levels, stress, quality of sleep, physical activity, and fruit and vegetable consumption within the digital platform. Stress, energy levels, and quality of sleep were entered on a rating scale from 0 to 10, physical activity was logged as steps/day, and fruits and vegetables were logged as servings per day.

At the beginning and the end of the study, self-reported questionnaires were administered to patients online. Anxiety and depression were assessed with the Hospital Anxiety and Depression Scale (HADS), which is a 14-item self-reported questionnaire. Responses were rated on 4-point Likert scales, where the score range for each subscale outcome is 0–21, and the lower scores indicate lower levels of anxiety or depression [20]. HF symptoms were assessed with the short version of the Kansas City Cardiomyopathy Questionnaire (KCCQ-12), which is a 12-item questionnaire designed to quantify HF-related symptom frequency, physical and social limitations, and quality of life impairment. Scores for the four domains range from 0 to 100, and the total summary score is the average of the scores of the four domains. Higher scores indicate a better health status [21]. The KCCQ-12 was completed by the participants at the beginning and the end of the 8-week study through an internet-based survey. At the end of the study, participants also completed a 5-item questionnaire about their experience and acceptability of SK-141, of which the answers were scored on seven-point Likert scales (see questions in Table 1).

**Table 1:** Participants’ experience and acceptability of the SK-141

Post-study question (score range^a^)	Mean (SD)
How likely are you to recommend this intervention to others? (1–7)	6.8 (0.4)
The educational videos were helpful (1–7)	6.2 (0.9)
The SidekickHealth mobile application was very user-friendly (1–7)	5.5 (1.4)
I feel like I am better able to manage my condition after completing the intervention (1–7)	4.9 (1.2)
The intervention has improved my life and well-being (1–7)	5.7 (0.9)

*SD* standard deviation

^a^Answers were scored on a seven-point Likert scale. Answers ranging from very unlikely and don't agree at all (=1) to very likely, very much agree (=7). Based on questionnaires completed by thirteen patients

### Statistical analyses

Demographic characteristics (age, gender) were summarized using frequency tables (*n* (%)) for categorical variables, while means and standard deviations were calculated for continuous data. While the study was not powered to detect statistical differences in clinical outcomes, we nevertheless chose to use paired *t*-tests to analyze any differences in key clinical outcomes and a *p* < 0.05 was deemed statistically significant. Statistical analyses were performed with SPSS IBM SPSS statistics version 27 (Build 1.0.0.1508 – 64-bit edition), and graphs were made with GraphPad Prism version 9.2.0.

## Results

### Participants, retention rate, and engagement

**Fig. 4:**
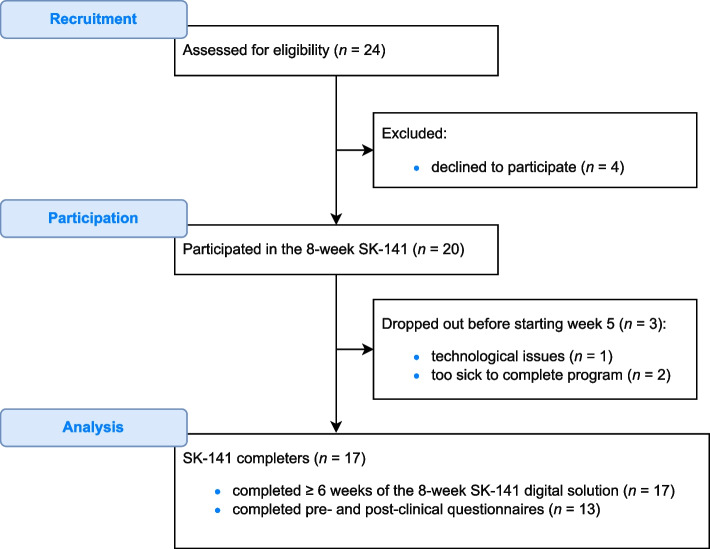
Flowchart of the study. The flowchart was generated with Diagrams software

Twenty-four HF patients attending the outpatient HF clinic were recruited for this study. Out of those, twenty (83%) agreed to participate and signed informed consent forms (Fig. 4). There were 16 (80%) men, and the mean age was 57.5 (range 38–79) years (Table 2). All participants had heart failure with a decreased ejection fraction. Three individuals dropped out, one because of having issues with their smartphone, and two due to illness requiring hospital admission, one of which was HF-related. Therfore, seventeen of the twenty participants completed the 8-week SK-141 program. Thirteen of those participants completed both the pre- and post-study questionnaires (Fig. 4), as four declined to answer the final questionnaires due to a variety of reasons. The participants performed an average of 53.9 activities within the digital platform during week 1 and 60.4 activities during week 8 (+11.9%, *p* = .59). The nurses in the HF clinic spent approximately one hour a day, on average, attending to the app.

**Table 2:** Demographic characteristics

	Started the SK-141 (***n*** = 20)	Completed the SK-141 (***n*** = 17)	Completed the pre- and post-study clinical questionnaires (***n*** = 13)
Age, mean (SD)	57.5 (11.7)	55.7 (11.3)	57.8 (10.5)
Male gender, *n* (%)	16 (80%)	13 (76.5%)	9 (69.2%)

*n* number of patients with data available, *SD* standard deviation

### Acceptability of the SK-141

At the end of the 8 weeks, thirteen of the participants completed a questionnaire about their experience and acceptability of the SK-141 (Table 1). They rated their experience regarding questions on whether they would recommend the solution to others at 6.8 on a scale of 1–7, whether the solution had improved their life and well-being (5.7 on a scale of 1–7), and whether it was user friendly (5.5 on a scale of 1–7) (Table 1).

### Changes in health-related factors

Based on the daily in-app reporting of energy levels, stress, quality of sleep, physical activity, and fruit and vegetable consumption, users reported an average improvement from week 1 until week 8 (Table 3). The improvements included a sharp increase in servings of fruit consumed (67.4%, *p* < .01) and a positive trend for improvements in energy levels (11.1%, *p* = .06) and quality of sleep (13.4%, *p* = .07), however, those changes and changes in steps and servings of vegetables from week 1 until week 8 were not statistically significant.

**Table 3:** Changes in health-related factors after 8 weeks of the SK-141

Variable (***n***)	Week 1 mean (SD)	Week 8 mean (SD)	***P-***value	Percentage change
Health-improving activities registered per week (17)	53.9 (28.3)	60.4 (55.0)	.589	+11.9%
Energy levels (15)	5.4 (1.9)	6.0 (1.6)	.060	+11.1%
Stress (15)	3.4 (1.9)	3.5 (2.2)	.867	+2.94%
Quality of sleep (15)	6.7 (1.9)	7.6 (1.3)	.072	+13.4%
Steps registered per week (15)	12054 [4305;23,466]	13085 [4826;30,757]	0.394^a^	+8.6%
Servings of fruit per week (11)	4.6 (4.2)	7.7 (5.7)	**.005**	+67.4%
Servings of vegetables per week (14)	8.4 (6.3)	9.8 (8.2)	.589	+16.7%

*n* number of subjects with data, *SD* standard deviation

Variables logged daily in, or automatically registered by, the digital platform

^a^*P*-value from a Wilcoxon signed-rank test

### Changes in clinical outcomes

All clinical outcomes (HADS and KCCQ-12), except ratings on the KCCQ-12 subscale Quality of Life (−1.8%, *p* = .79), improved from week 1 until week 8 (Table 4), although the changes were not statistically significant. On the HADS scale, both scores on the subscales Anxiety (−19.2%) and Depression (−13.6%) were reduced, with scores for Anxiety significantly improved (*p* < .05). For the KCCQ-12 scale, most notably, ratings on the subscale Symptom Frequency were greatly improved (+41.0%), although this was not statistically significant (*p* = .10), likely due to the small sample size. Participants reported significantly fewer limitations by their experience of one of the key symptoms of HF: shortness of breath (pre-score: 2.6 vs. post-score: 3.9, *p* < .05), which is a variable on the subscale Symptom Frequency (KCCQ-12).

**Table 4:** Changes in clinical outcomes after 8 weeks of SK-141

Outcome variable	Baseline mean (SD)	Post-SK-141 mean (SD)	***P-***value	Percentage change
HADS				
Anxiety	5.2 (3.9)	4.2 (3.5)	**.033**	-19.2%
Depression	5.8 (4.0)	5.1 (3.8)	.293	-13.6%
KCCQ-12				
Physical limitation	57.1 (23.3)	58.3 (19.2)	.711	+2.1%
Symptom frequency	29.3 (26.8)	41.4 (27.5)	.103	+41.0%
Quality of life	55.8 (30.0)	54.8 (29.6)	.794	-1.8%
Social limitation	52.6 (23.7)	54.5 (23.5)	.553	+3.6%
Total scale summary	48.7 (12.9)	52.3 (12.3)	.153	+7.4%

*HADS* Hospital Anxiety and Depression Scale, *KCCQ-12*, Kansas City Cardiomyopathy Questionnaire, *n* number of subjects with data, *SD* standard deviation

## Discussion

We performed a small study in HF patients to assess the acceptability, and trends in the clinical effectiveness of a digital solution for HF self-care that provides remote symptom monitoring, support for lifestyle changes and self-care, and a messaging platform for secure healthcare and patient communication. The digital solution was perceived as user-friendly, which demonstrates reasonable user acceptability and supports the feasibility of the SK-141. The refusal rates for participation were low, while both the retention and engagement rates were high. In addition, although this was not a key goal of the study, improvements for six out of seven clinical outcomes measured were found. However, only the improvements in HF patients' anxiety and shortness of breath were statistically significant, which nevertheless was encouraging given the very small sample size. In addition, all-but-one self-reported variables logged within the digital platform appeared to improve over time.

Digital solutions offer a unique capacity for remote symptom monitoring and patient support, providing real-time access from any location while potentially improving resource utilization and decreasing the overall cost of care [13]. The increasing demand for self-care technologies has further intensified since the onset of the COVID-19 pandemic [13]. In cardiology, remote monitoring of cardiac implantable electronic devices, such as pacemakers and implantable cardioverter defibrillators, has already been shown to reduce all-cause mortality as well as the composite endpoint of all-cause mortality or HF-related hospitalization [22]. The use of digital solutions to improve self-care among HF patients is a relatively new field, and in the small studies that have been done so far not all types of solutions were found to be effective [16]. However, digitally delivered patient support appears to benefit HF self-care and prevent hospital (re)admissions as previous studies, in line with the findings from this study, have demonstrated very promising positive trends [23]. The new digital solution (SK-141) that was used in this study was specifically developed to improve the self-care of HF patients. In contrast to other solutions and remote medical care systems, in the design of SK-141 many features -such as gamification of tasks- were incorporated to enhance patient engagement and adherence, with the aim to ultimately reduce HF morbidity and mortality.

There may be certain barriers to the access and the use of online health information that can result from the presentation of content and poor technical adaptability to various digital solutions. Socioeconomic status may also affect the possibility of using digital solutions although this was not addressed specifically in this study. A number of possible solutions may increase the digital health literacy of patients. Among those is walking the patient through how to use a remote patient monitoring device which is likely to increase the odds that patients continue to use. That demonstration should also emphasize privacy and security, two key factors for patients considering health IT adoption. Others may include a high emphasis on a user-friendly interface and potentially incorporating a relevant third party from the family in the program. Efforts are already underway with the SK-141 digital solution in this regard. Mobile telephone apps may hold potential for digital health services tailored to people with low health literacy [24]

The key target groups for the use of digital solutions remain to be determined. While an application such as this one, SK-141, may be a welcome addition for patients with active chronic disorders like heart failure, even in those with end-stage disease, it is also perceivable that patients with stable diseases may also benefit. In such patient groups, digital follow-up could even to some extent replace visits to the outpatient clinic, as information is obtained via remote monitoring and feedback provided in addition to a platform for questions. As human resources in health care are becoming more and more scarce, task shifting in terms of more remote than onsite follow-up for stable individuals may need to be considered. For this to happen, however, cost-effectiveness in addition to the issue of reimbursement or dedication of funds in a government funded health care system need to be addressed. Thus, while significant challenges remain in digital medicine, the effectiveness and potential to provide more targeted care need to be carefully studied. Retail and banking are two areas which have been revolutionized by digital applications. This has demonstrated that long-standing societal traditions can be changed with online solutions and we believe that they may eventually have a similar effect on healthcare delivery.

There are a number of potential adverse effects of digital medicine. These include patient-related factors and possible negative effects on health care systems. Many of these digital solutions already in use were taken up without research evidence about their clinical efficacy or the best practices for their use. Beyond this, other potential digital medicine issues include implications for health equity, the possibility of information overload, uncertainty about which digital solutions to use in what patient situations, and implementation standards for integrating digital tools into routine clinical operations and electronic health records systems. These are big challenges and underscore the need for good research in digital medicine.

A clear limitation of the current study is the small sample size, with only 20 participants, which makes it less likely to detect significant clinical changes. Also, the study included mostly men and the upper age limit was 80 years. Older individuals are less likely to use smartphones or tablets and thus we set this limit arbitrarily. Future studies will, however, also need to include individuals of all ages and include a more balanced gender ratio. There were more males in this study, which was a reflection of this demographic in the HF clinic. Yet another limitation is the short duration of the study, as 8 weeks may be too short to improve lifestyle and clinical outcomes. It must, however, be emphasized that the study was first and foremost designed to assess the feasibility and patients' acceptance of the approach and not to detect clinically meaningful differences, and therefore the study was not powered as such. The acceptability of the digital intervention, the high retention and engagement rates, and promising trends in the clinical outcomes are in our opinion very encouraging. This suggests that an adequately powered, randomized-controlled study with a longer duration should be undertaken to obtain more robust results and test the hypothesis that the SK-141 digital solution on top of standard medical therapy results in improved quality of life and better clinical outcomes among patients in the outpatient HF clinic. In the future studies also need to include patients with different subtypes of heart failure.

Based on the variety of outcomes examined, the range of effect sizes was utilized

for sample size determination for a larger randomized trial. Based on the feasibility study, the largest effect size was for symptom frequency and the smallest effect size was for the quality of life (as measured by the KCCQ-12). Assuming a medium effect size was reasonable for most of the key outcomes (e.g., symptom frequency, and KCCQ-12 overall). In order to increase sensitivity for smaller effect sizes and small clinically important changes (five points) as reported for the KCCQ scale, with 80% power, two groups (SK-141 and standard care versus standard care only), four time points (0, 3 months, 6 months, 12 months), 0.5 correlation between repeated measures, and assuming 80% retention rate, a total of at least 174 participants (split equally across conditions) are needed. Feedback and insights from this feasibility study are used to further improve the features and content of the solution while planning for a randomized-controlled study of a longer duration.

In conclusion, this small feasibility study showed that the SK-141 was easy to use and acceptable to patients, had a high retention rate and there was a trend towards a positive impact on their health and well-being. These findings are promising and support conducting a more extensive, adequately powered, randomized-controlled study comparing the effects of standard HF care to enhanced care incorporating this new digital solution.

## Supplementary Information

**Additional file 1: Table S1.** Changes in health-related factors after 8-weeks of the SK-141.

**Additional file 2: Table S2.** Changes in clinical outcomes after 8-weeks of SK-141.

## Data Availability

The datasets used and/or analyzed during the current study are available from the corresponding author on reasonable request.

## Abbreviations

HADS: Hospital Anxiety and Depression Scale
HF: Heart failure
KCCQ-12: Kansas City Cardiomyopathy Questionnaire, short version
SD: Standard deviation
