# Supplementary material for: Improving outpatient care for heart failure through digital innovation: a feasibility study
Source: Pilot Feasibility Stud. 2022 Nov 30;8:242. doi: 10.1186/s40814-022-01206-w (PMC9709356; doi:10.1186/s40814-022-01206-w)
Supplement: Supplementary file 1 — Additional file 1: Table S1. Changes in health-related factors after 8-weeks of the SK-141. [file 40814_2022_1206_MOESM1_ESM.docx]

## **Table S1. Changes in health-related factors after 8-weeks of the SK-141**

|  | **Week 1** | **Week 8** | **P-value** | **Percent change** |
| --- | --- | --- | --- | --- |
| **Variable (*n*)** | **Median (25^th^;75^th^)** | **Median (25^th^;75^th^)** |  |  |
| Health-improving activities registered per week (17) | 52.0(38.0;75.0) | 42.0 (37.0;61.0) | 0.981 | -19.2% |
| Energy levels (15) | 6 .0(4.0;7.0) | 6.0 (5.0;7.5) | **0.045** | 0.0% |
| Stress (15) | 3 .0(2.0;5.0) | 3.0 (2.0;5.0) | 0.71 | 33.3% |
| Quality of sleep (15) | 7.0 (5.0;8.0) | 7.8 (7;8.8) | 0.077 | 11.4% |
| Steps registered per week (15) | 12054 (4305;23466) | 13085 (4826;30757) | 0.394 | 8.6% |
| Servings of fruit per week (11) | 3.0 (1.0;8.0) | 6.0 (3.0;11.0) | **0.003** | 100.0% |
| Servings of vegetables per week (14) | 7.0 (6.0;9.0) | 7.0 (2.0;16.0) | 0.593 | 0.0% |

*n*, number of subjects with data; **(25^th^;75^th^)**,percentiles.

Variables logged daily in, or automatically registered by, the digital platform.
