# Supplementary material for: Improving outpatient care for heart failure through digital innovation: a feasibility study
Source: Pilot Feasibility Stud. 2022 Nov 30;8:242. doi: 10.1186/s40814-022-01206-w (PMC9709356; doi:10.1186/s40814-022-01206-w)
Supplement: Supplementary file 2 — Additional file 2: Table S2. Changes in clinical outcomes after 8-weeks of SK-141. [file 40814_2022_1206_MOESM2_ESM.docx]

**Table S2. Changes in clinical outcomes after 8-weeks of SK-141**

|  | **Baseline** | **Post-SK-141** | **P-value** | **Percent change** |
| --- | --- | --- | --- | --- |
|  | **Median (25^th^;75^th^)** | **Median (25^th^;75^th^)** |  |  |
| **Outcome variable** |  |  |  |  |
| HADS |  |  |  |  |
| Anxiety | 5.0 (2.0;7.0) | 4 .0(1.0;6.0) | **0.037** | -20.0% |
| Depression | 4.0 (3.0;9.0) | 4 .0(3.0;6.0) | 0.275 | 0.0% |
| KCCQ-12 |  |  |  |  |
| Physical Limitation | 50.0 (41.7;75) | 58.3 (50.0;75) | 0.806 | 16.6% |
| Symptom Frequency | 18.8 (8.3;47.9) | 37.5 (18.8;58.3) | 0.058 | 99.5% |
| Quality of Life | 50.0 (37.5;75) | 50 (37.5;75) | 0.687 | 0.0% |
| Social Limitation | 50.0 (41.7;66.7) | 66.7 (41.7;66.7) | 0.412 | 33.4% |
| Total Scale Summary | 46.9 (40.1;57.3) | 52.1 (44.3;57.3) | 0.173 | 11.1% |

HADS, Hospital Anxiety and Depression Scale; KCCQ-12, Kansas City Cardiomyopathy Questionnaire; *n*, number of subjects with data; **(25^th^;75^th^)**,percentiles
